# Rice husk derived Aminated Silica for the efficient adsorption of different gases

**DOI:** 10.1038/s41598-020-76460-0

**Published:** 2020-11-11

**Authors:** Rashed S. Bakdash, Isam. H. Aljundi, Chanbasha Basheer, Ismail Abdulazeez

**Affiliations:** 1 Department of Chemistry, King Fahd University of Petroleum and Minerals, Dhahran, 31261 Saudi Arabia; 2 Department of Chemical Engineering, King Fahd University of Petroleum and Minerals, Dhahran, 31261 Saudi Arabia

**Keywords:** Biotechnology, Environmental sciences, Chemistry, Energy science and technology, Engineering

## Abstract

In this present work, we successfully prepared aminated silica (ASiO_2_) from rice husk ash (RHA) and functionalized with 3-aminopropyltriethoxysilane (APTES). Physical and chemical properties of the synthesized material were investigated by various techniques SEM–EDX, XPS, FTIR, TGA. The surface area of RHA was 223 m^2^/g, while for ASiO_2_ was 101 m^2^/g. Molecular level DFT calculations revealed that the functionalization of ASiO_2_ resulted in a significant decrease in the HOMO–LUMO energy gap, a reduction in hardness, and a consequent increase in charge transfer characteristics. The adsorption behavior at low pressure (1 atm.) of aminated silica on different gases CO_2_, CH_4_, H_2_, and N_2_ at temperatures 77, 273, 298 K was studied. The adsorption of hydrogen was reported for the first time on aminated silica with an excellent adsorption capacity of 1.2 mmol/g. The ASiO_2_ exhibited excellent performance in terms of gas separation in binary mixtures of CO_2_/CH_4_, CO_2_/N_2_ and CO_2_/H_2_ at 273, and 298 K, respectively. The catalyst further exhibits high stability during three cycles with less than 10% variation in the separation capacity.

## Introduction

The flexibility of silica-based mesoporous materials enables their functionalization with other materials, such as metal nanoparticles, or rare-earth elements^1^. Mesoporous silica has an advantage of high surface area and pore volume, high stability, unique pore structure characteristics, and uniform pore size distributions. It has been widely used in wastewater treatment, air purification, thermal insulation systems, oxygen, and humidity sensors and battery electrodes applications^2^. Other applications include as gas adsorbent due to its controllable pore size and volume. The functional groups present on the surface of mesoporous silica contributes significantly to its gas adsorption–desorption capacity and its interactions with other materials^1,3^.

The rising average temperature of the earth as a result of an increased presence of greenhouse gases such as CO2 have raised a lot of concerns in recent years. CO_2_ is considered one of the major contributors (up to 60%) to total greenhouse gas emissions. To reduce the impact of CO_2_ gas, the CO_2_ Capture, Storage and Utilization technologies were introduced. However, the sustainability of these technologies remain of concern due to the cost implications^4^. The captured CO2 can be converted into valuable chemicals such as methane which is considered a green source of power generation in households^4,5^. The purification and upgrade of biogas (CH_4_, CO_2_, and N_2_) to meet pure natural gas (CH_4_) (pipeline quality) to avoid the corrosion can be achieved by effective cheap techniques to separate CO_2_ and N_2_ gas, such as absorption^6^, cryogenic separation^7^, membrane separation^8^, distillation^9^ and adsorption^10^. More specifically, solid adsorbents are more effective and less expensive than liquid absorbents like amine solvents, which have some restrictions due to their volatility, tendency to cause corrosion and high-energy consumption, especially in long-term application^4,5,11–14^. CO_2_ capture as a physisorption process can be achieved by using activated carbon, silica gel, molecularly imprinted adsorbents, metal–organic frameworks (MOFs), and mesoporous molecular sieve^2^. The development of mesoporous materials have attracted more attentions in recent years for CO_2_ adsorption due to their high porosity, high gas diffusibility, and large pore volumes. Moreover, the separation capacity and selectivity of CO_2_ on mesoporous materials are not good enough. So, the improvement of these materials is highly attractive^15^. The functionalization of mesoporous adsorbent with various type of amines will enhance the interactions with CO_2_ compared with microporous materials due to the formation of ammonium carbamates and carbonates reversibly at moderate temperature^16,17^. Amine functionalization results in highly efficient CO_2_ capture at low temperature due to the strong interaction between the CO_2_ molecules and the porous structure, resulting in high gas diffusion through the adsorbent^18^.

Kumar et al.^19^, reported the functionalization of commercial silica with polyethyleneimine and 3-aminopropyltriethoxysilane (APTES) for CO_2_ capture. The amine functional group drastically enhanced the adsorption capacity of CO_2_, and the results revealed that the presence of higher amine contents resulted in higher CO_2_ uptake by the adsorbent. Similarly, rice husk derived mesoporous silica functionalized with various amines have been investigated for CO2 adsorption at different temperatures. The results showed that the branched amines exhibits higher CO2 adsorption capacities compared to straight-chain amines^20,21^. Zeng and Bai have reported a low-cost mesoporous silica with large pore volume impregnated with tetraethylenepentamine for efficient CO_2_ adsorption with a maximum CO_2_ uptake up to 173 mg/g^22^.

Recently, hydrogen adsorption on mesoporous silica SBA-15 was reported^23^. SBA-15 with a high surface area up to 3274 m^2^/g interestingly exhibited a low sorption capacity towards hydrogen at 77 K and 298 K^24^. To further improve the performance, mesoporous silica was functionalised with aluminium and platinum^25^ and titanium nanoparticles^26^. The presence of nanoparticles enhances the hydrogen uptake by two-folds. As the size of the nanoparticles decreases, the adsorption capacity increases. However, not many studies have reported the adsorption and separation of binary gases mixtures other than hydrogen.

In this study, rice husk-derived aminated silica (ASiO_2_) was prepared and investigated for the adsorption and separation of a mixture of gases (CO_2_, CH_4_, H_2_, and N_2_) at low-pressure (1 atm.) and varying temperatures (298, 273 and 77 K).

Rice husk ash, RHA was extracted from rice husk and used to prepare the amine-modified silica^2^. The modification of RHA was conducted using APTES and confirmed by XRD, BET, FTIR, TGA, and SEM. The modified silica was used to study the adsorption of CO_2_. Molecular level DFT simulations were conducted to understand the underlying mechanistic insights into the role played by amine functional groups in the enhancement of the adsorption capacity of mesoporous silica. The present study demonstrates the role of amine presence in the enhancement of the adsorption and gas separation capacity of mesoporous silica materials, and would open up new horizons in the development of highly effective sorbents for CO2 capture.

## Experimental

### Chemicals and materials

Rice husk obtained from a rice mill from India and gained as a by-product of rice was employed as the initiating material without any pretreatment. Sulfuric acid (H_2_SO_4_, 95%) was purchased from Cromoline for washing and cleaning of RH. RHA (SiO_2_) was extracted from RH by heat treatment. Hydrochloric acid (37%) for acidification of RHA and anhydrous toluene (99.8%) as a solvent was bought from Sigma-Aldrich (St. Louis, USA). 3-aminopropyltriethoxysilane (APTES) was purchased from Sigma-Aldrich (St. Louis, USA).

### Preparation of rice husk ash (RHA)

The rice husk was washed with double distilled water (DDW) and 1.0 M sulfuric acid to remove all impurities, then dried in the oven overnight at 100 °C. Thereafter, the dried clean RH was ashed in the furnace at 700 °C for 6 h.

### Preparation of aminated silica (ASiO_2_)

RHA was soaked with 1.0 M HCl for 2 h, then washed with double distilled water, then dried in the oven at 100 °C overnight. 5 g of the acidified silica was added in 50 mL of dry toluene with 5 mL of APTES while stirring for 24 h at 120 °C. The resultant product was washed with DDW and dried overnight in the oven at 100 °C^27^. The prepared aminated silica was called (ASiO_2_).

### Material characterization

#### Surface morphology

Scanning electron microscopy (SEM) (Lyra3 TESCAN) was performed to investigate the surface of the RHA and ASiO_2_ materials. Energy-dispersive X-ray spectroscopy (EDX) scan was conducted to examine the existence of the functional groups on the surface of prepared materials. X-ray photoelectron spectroscopy (VG Scientific ESCALAB Mk (II) spectrometer using a non-monochromatic Al source (Kα, 1486.6 eV) was used to confirm the elemental composition on the sorbent surface.

#### BET surface area

For the Brunauer–Emmett–Teller (BET) surface area measurements, 0.1 g of the sample was loaded in a BET quartz tube at 200 °C for two hours in a vacuum. Nitrogen adsorption isotherms were obtained by Quantachrome Autosorb iQ-MP-C-XR. To measure the surface area and average pore size of RHA and ASiO_2_, the BET equation was used^28,29^.

#### Fourier transform infrared spectroscopy (FTIR)

The FTIR spectra were acquired by employing a Nicolet 6700 FT-IR (Thermo Electron Corporation). Potassium bromide was utilized to prepare a sample pellet, and the spectra were achieved in the range of 4000–400 cm^−1^ with a resolution of 4 cm^−1^ by the assemblage of 32 scans.

#### X-ray diffraction (XRD) and Thermal gravimetric analysis (TGA)

In order to get the XRD pattern of RHA and ASiO_2_ adsorbent were acquired by the Rigaku Miniflex II desktop X-ray diffractometer (tube output voltage 30 kV) at a scan rate of 2.5º min^−1^ from 3 to 100º.

Thermal stability was investigated using SDT-Q 600 TGA Instrument (New Castle, DE) with a flow rate of nitrogen at 75 ml/min and the maximum temperature of 1000 ◦C with a heating rate of 10 ◦C /min.

### Gas adsorption

The CO_2_, H_2_, CH_4_, and N_2_ gases were adsorbed by RHA and ASiO_2_ using Quantachrome Autosorb iQ-MP-C-XR. The experimental procedure of adsorption is as follows: initially, about 30–100 mg of each sample was loaded for evacuation to remove all moisture and gases up to 200 °C under an N_2_ and He atmosphere for 3.5 h. Then, the sample was loaded for adsorption of the gases at different temperatures under high-pressure. At the end of the adsorption studies, the system was switched to very low pressure for desorption of adsorbed gas. At this stage, one adsorption–desorption circle was performed. To investigate the reversibility of the adsorbent, three adsorption–desorption cycles were conducted^4^.

### Computational methods

Geometry optimization and vibrational frequency calculations were carried out on simplified lowest energy isomer of silica cluster, Si_4_O_6_^30^, APTES and APTES functionalized silica using density functional theory (DFT) approach, with the exchange–correlation treated using the hybrid GGA exchange functional of Becke^31^ and the PW91 correlation functional of Perdew and Wang^32^, BPW91 and 6-31G basis set. Full structural optimizations were carried out to the minima, and vibrational frequency analysis showed the absence of imaginary frequencies. Total energies of the natural bonding orbitals of the adsorbents, bond properties, and their relative binding distances with the gases were computed. Variations in temperature adsorption studies were carried out using temperature command in the input files before running the calculations. Adsorption energies (Δ*E*_ads_) of the gases on the adsorbents were estimated using the eqs:

$$\Delta E_{ads} = -\Delta E_{binding} $$

$$\Delta E_{binding} = E_{ads/gas } - \left( {E_{ads} + E_{gas} } \right) $$

where *E*_ads/gas_ represents the free energy of the adsorbent-gases complex and *E*_ads_, *E*_gas_ the free energies of the isolated adsorbents and the gases, respectively. All calculations were carried out using the Gaussian 09 program.

## Results and discussion

### Surface morphology

**Figure 1:**
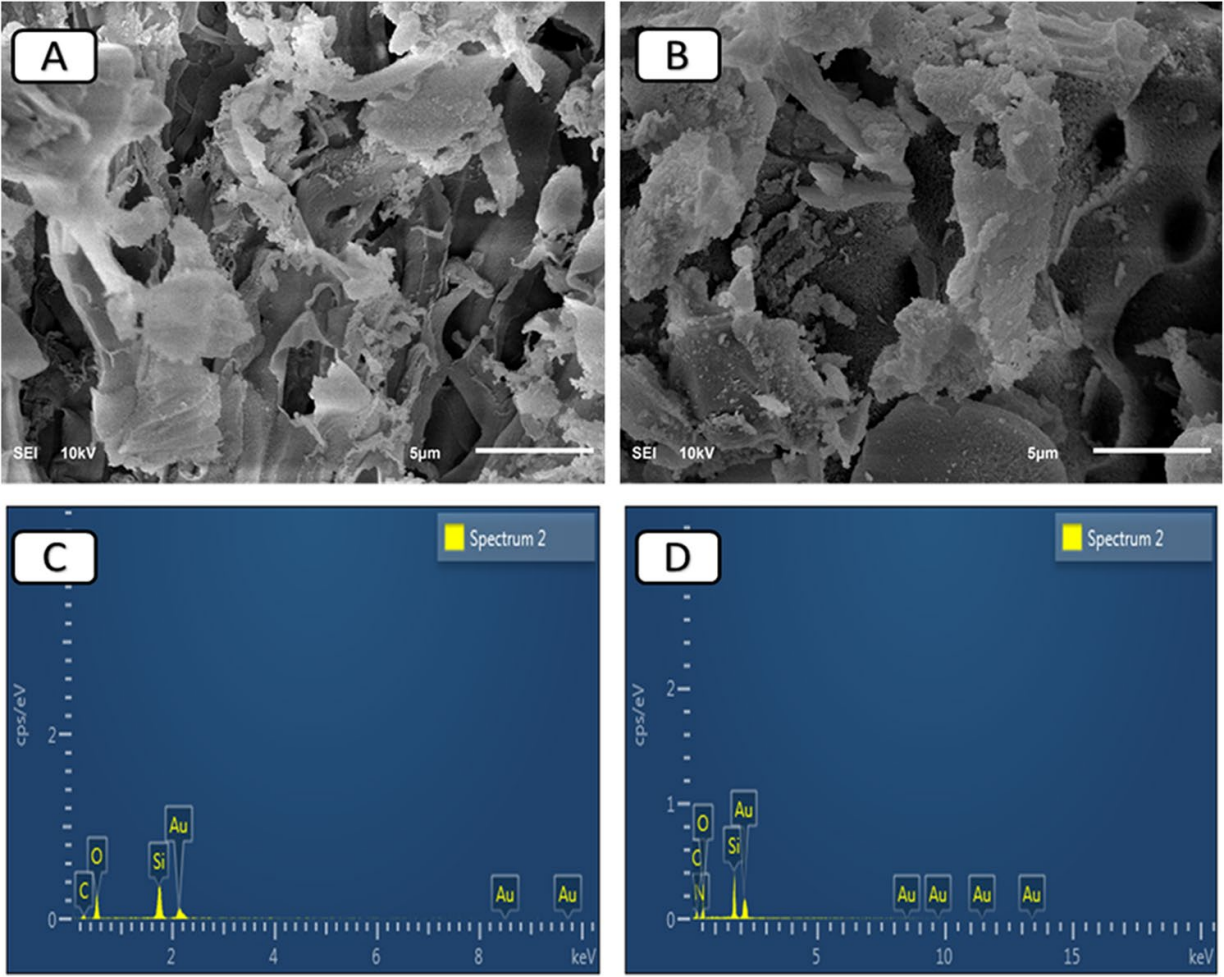
SEM micrographs and EDX analysis of RHA (**A**, **C**) and ASiO_2_ (**B**, **D**).

The SEM–EDX monographs of RHA and ASiO_2_ are shown in Fig. 1. There is no significant difference in the morphology of the surface before (Fig. 1A) and after the functionalization of RHA with APTES (Fig. 1B). The surface of ASiO_2_ became darker due to the presence of the amine group on the surface^19^. The EDX analysis of RHA (Fig. 1C) shows a significant amount of oxygen and silicon of 43.2% and 37.2%, respectively. On the other hand, the functionalization of RHA with APTES was confirmed by EDX analysis (Fig. 1D) and showed a content of C, O, Si, and N of 27.4%, 38.3%, 31.1%, and 3.2%; respectively.

**Figure 2:**
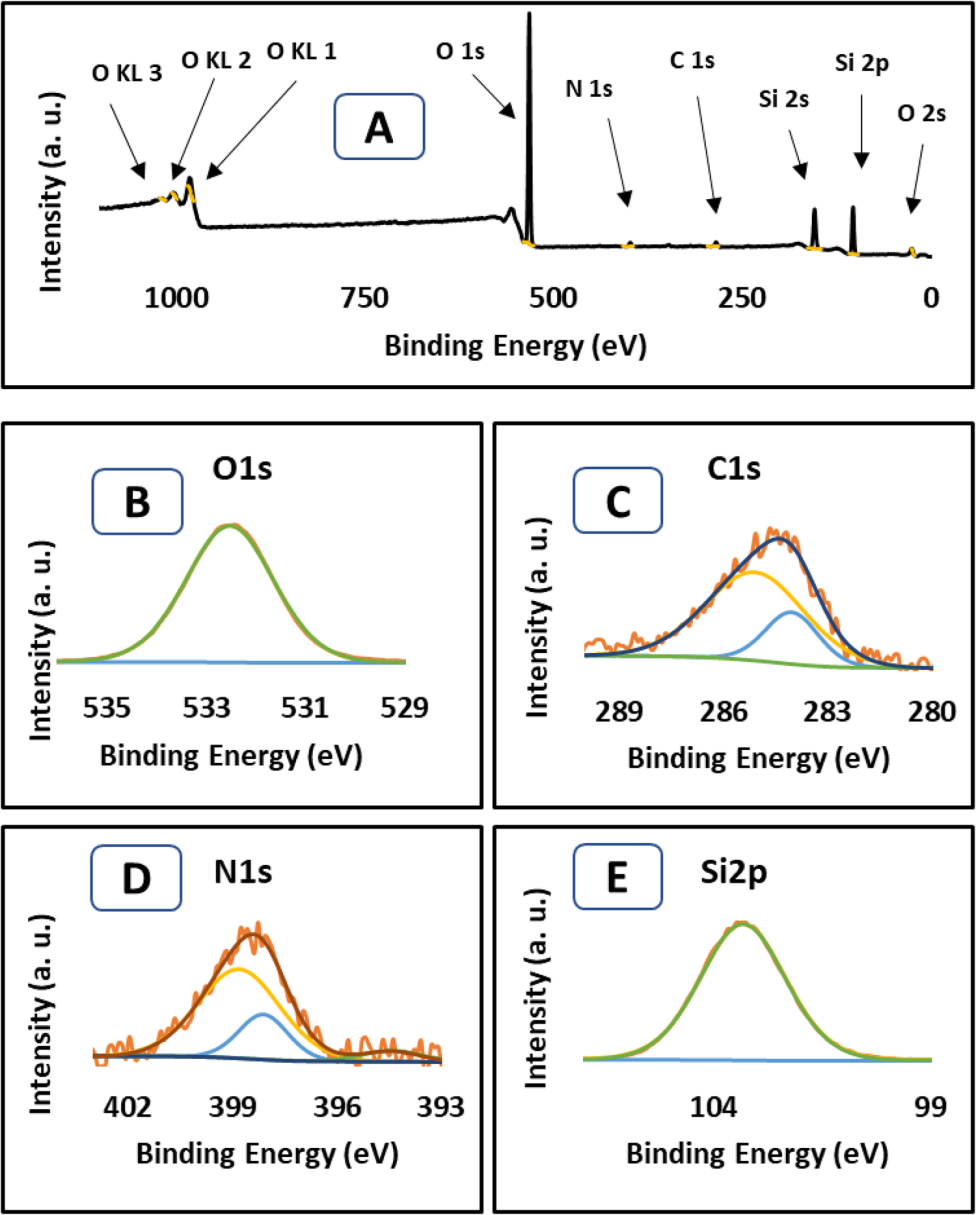
XPS analysis of ASiO_2_, (**A**) broad scan spectrum, the high-resolution spectrum of (**B**) O 1s, (**C**) C 1s, (**D**) N 1s, and (**E**) Si 2p.

The XPS full scan of ASiO_2_ (Fig. 2A) demonstrates the prominent elemental variation in the structure, which confirms the treatment of RHA with APTES. This data showed the introduction of nitrogen and carbon into the structure and is compatible with the EDX data. The high-resolution carbon spectra (Fig. 2C) showed the peak at ∼ 284 eV and ∼ 285 eV that can be allocated to C–C and C–N or C–O bond, respectively^33^. In the case of N (1s) spectra (Fig. 2D), the peak at ∼ 398 eV corresponds to NH_2_ while the peak at ∼ 399 eV corresponds to NH_3_^+^^34^. Figure 2B showed the high-resolution scan of O (1s) with major peak and binding energy at ∼ 532.5, which is related to ethoxy group bond O–C^33^. The data in (Fig. 2E) demonstrates the high-resolution XPS band of Si (2p). The deconvoluted binding energy peaks at ∼ 103.3 eV can be ascribed to the Si–O bond^35^. For a comparative purpose, XPS data for SiO_2_ is provided in the supplementary Fig. 1S.

### Pore size distribution measurements

**Figure 3:**
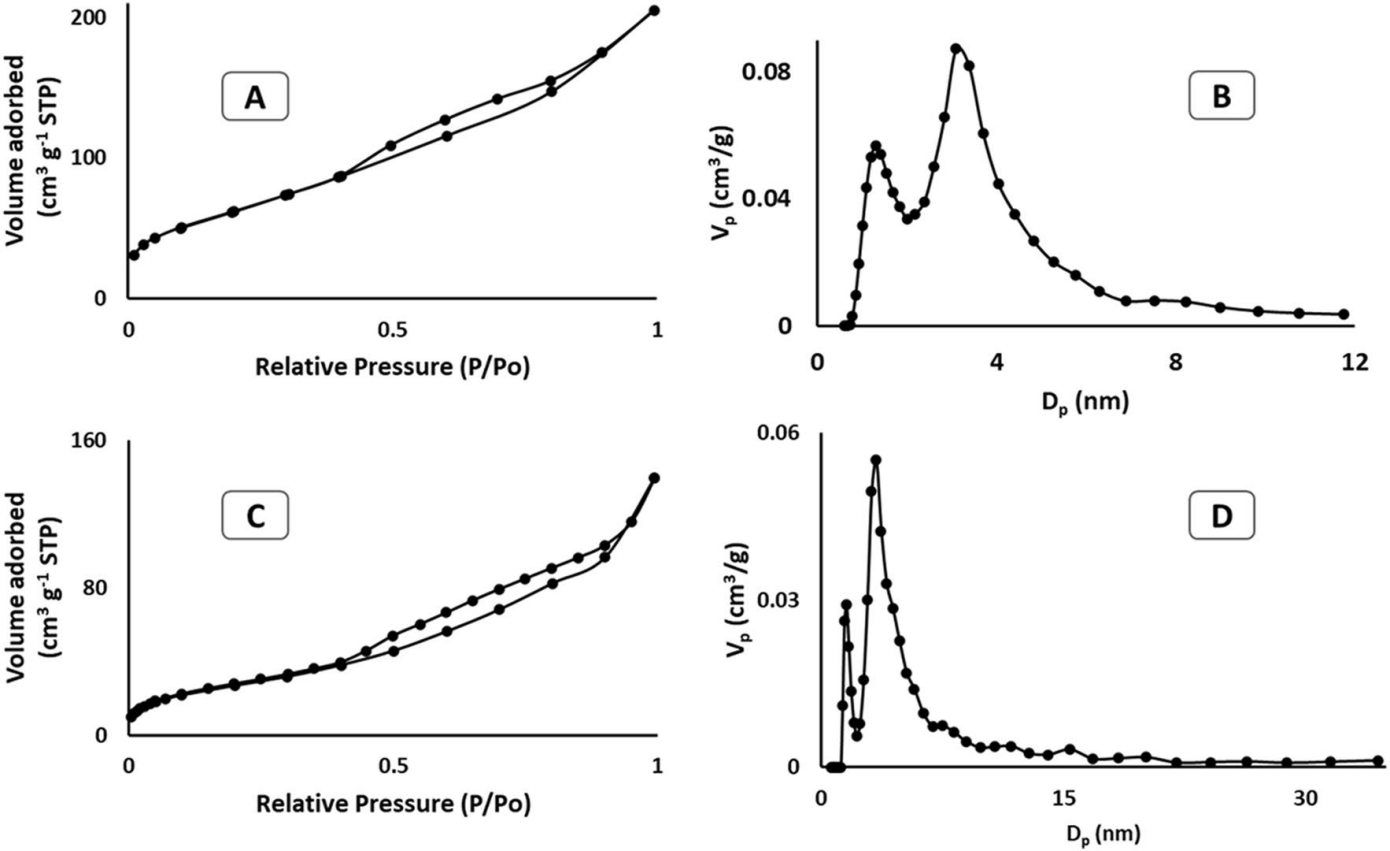
Nitrogen adsorption isotherm (**A**) and Pore size distribution (**B**) of RHA and N_2_ isotherm (**C**) and Pore size distribution (**D**) of ASiO_2_.

Nitrogen adsorption was utilized to measure the surface area and demonstrate the pore features of the produced materials. Figure 3 shows the N_2_ adsorption isotherm of RHA and ASiO_2_ at 77 K. The adsorbed volume of nitrogen on RHA uninterruptedly upsurged but did not attain a plateau close to the relative pressure (P/P_o_) of 1.0, inferring the existence of mesopores. In the case of ASiO_2_, the adsorption capability is lower at low pressure as compared to RHA confirming the loading of the amino group on the surface. The surface area, pore diameter, and pore volume of RHA and ASiO_2_ are shown in Table 1.

**Table 1:** Structural properties of RHA and ASiO_2_.

Sample	S_BET_ (m^2^/g)	V_p_ (cm^3^/g)	D_p_ (nm)
RHA	220	0.248	3.385
ASiO_2_	100	0.179	3.099

The pore size distribution of RHA is from 3.3 nm to 12 nm with a pore volume of 0.25 cm^3^/g, confirming the presence of mesopores in RHA (Fig. 3). However, the porous structure of ASiO_2_ (Fig. 3) mainly had mesopores distributed up to 12 nm with a mean diameter of 3.0 nm and a pore volume of 0.18 cm^3^/g. The decrease in pore size and pore volume of ASiO_2_ is mainly due to pore filling with APTES^19^.

### Thermal gravimetric analysis

**Figure 4:**
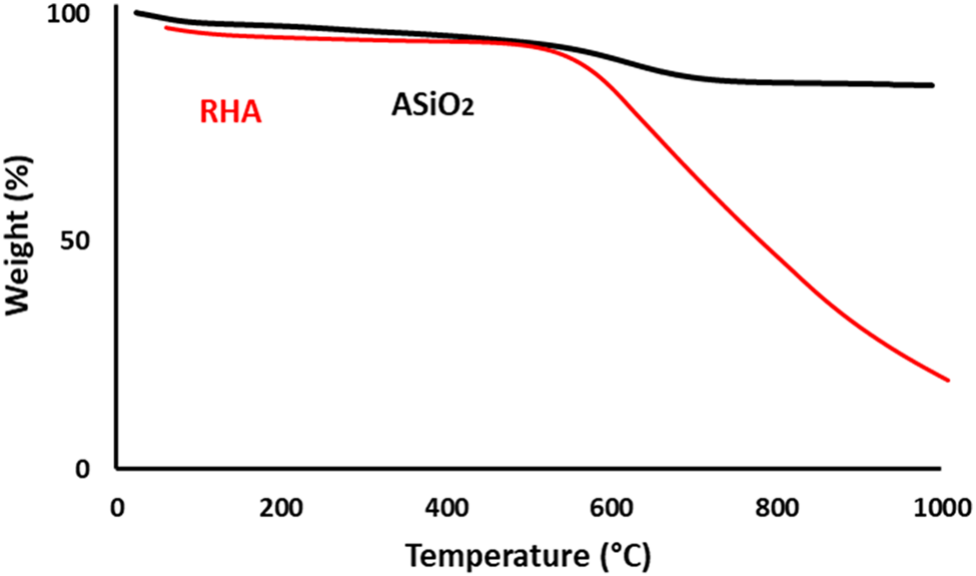
TGA analysis of RHA and ASiO_2_.

The thermal stability of the silica-based materials was investigated with TGA analysis (Fig. 4). When the temperature reached 1000 °C, 5% of RHA and 85% of the ASiO_2_ materials remained as a residual solid. The analysis revealed that there are three zones in the TGA curves of each adsorbent. In the case of amine-modified silica, the adsorbent shows a little decrease of weight in the range of 50–600 °C due to the removal of guest molecules and moisture. Further, an increase in temperature up to ~ 800 °C showed a small change in the weight of ASiO_2_ that is referred to as the degradation of the amine functional groups. Above 800 °C, no significant weight loss was observed, which is consistent with the findings of other researchers^2^. While in the case of RHA, the first weight loss up to 400 °C was due to the release of moisture and other adsorbed gases. The second region with a significant decrease in weight extended up to 1000 °C, which is related to the decomposition and co-condensation of silica components^1^.

### Spectroscopic characterizations

**Figure 5:**
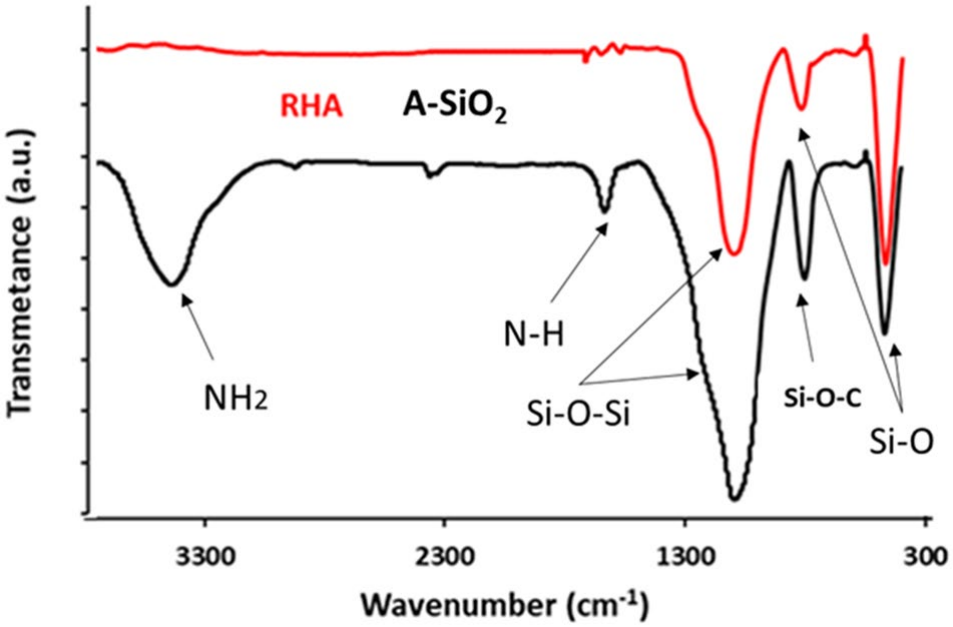
FTIR analysis of RHA and ASiO2.

FTIR spectra of the silica-based materials are presented in Fig. 5, and the characteristic absorption bands can be observed for RHA and ASiO_2_. The absorption bands of RHA at 466 cm^−1^ is ascribed to Si–O distortion, 800 cm^−1^ corresponds to Si–O bending, and Si–O–Si stretching appeared at 1078 cm^23^. After modification of RHA with APTES, the peak at 790 cm^−1^ represent Si–O–C stretching vibration, while an additional peak appeared at 3420 cm^−1^ which corresponds to the silanol hydroxyl group (Si–OH). The peak at 3360 and 3240 cm^−1^ are represents the -NH_2_ vibrational stretching. The peak at 2920 cm^−1^ represents the C–H stretching of –CH_2_ while the peak at 1630 cm^−1^ corresponds to the N–H bending of the amine functional group^1,19,36,37^.

**Figure 6:**
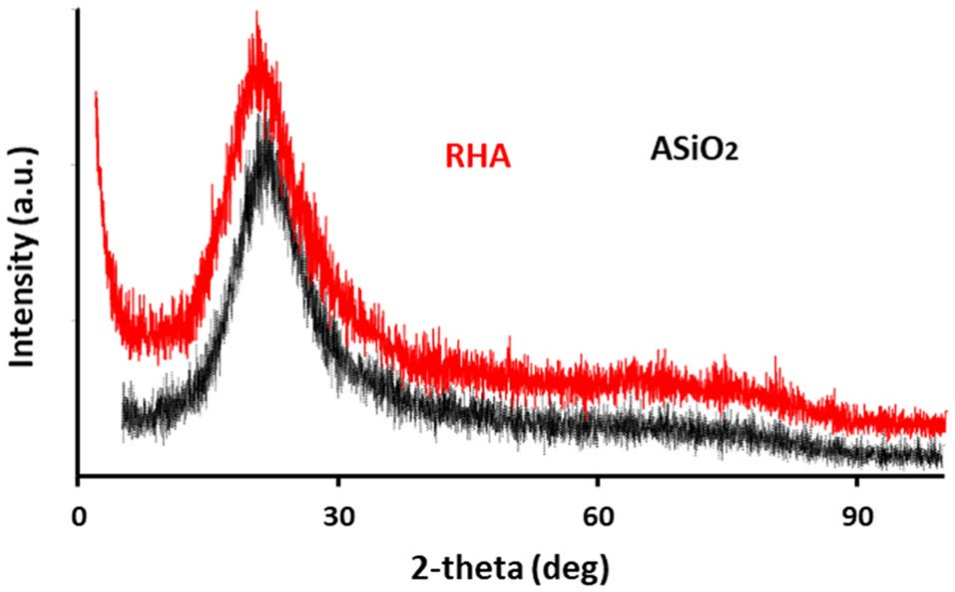
XRD patterns of RHA and ASiO_2_.

Figure 6 displays X-ray diffraction (XRD) patterns of RHA and ASiO_2_. A broad peak appeared at around 2θ = 25°, which represents the semi-amorphous nature of the prepared materials. The XRD analysis showed that the structural properties of the prepared ASiO_2_ were maintained after modification.

### Gas adsorption behavior

**Figure 7:**
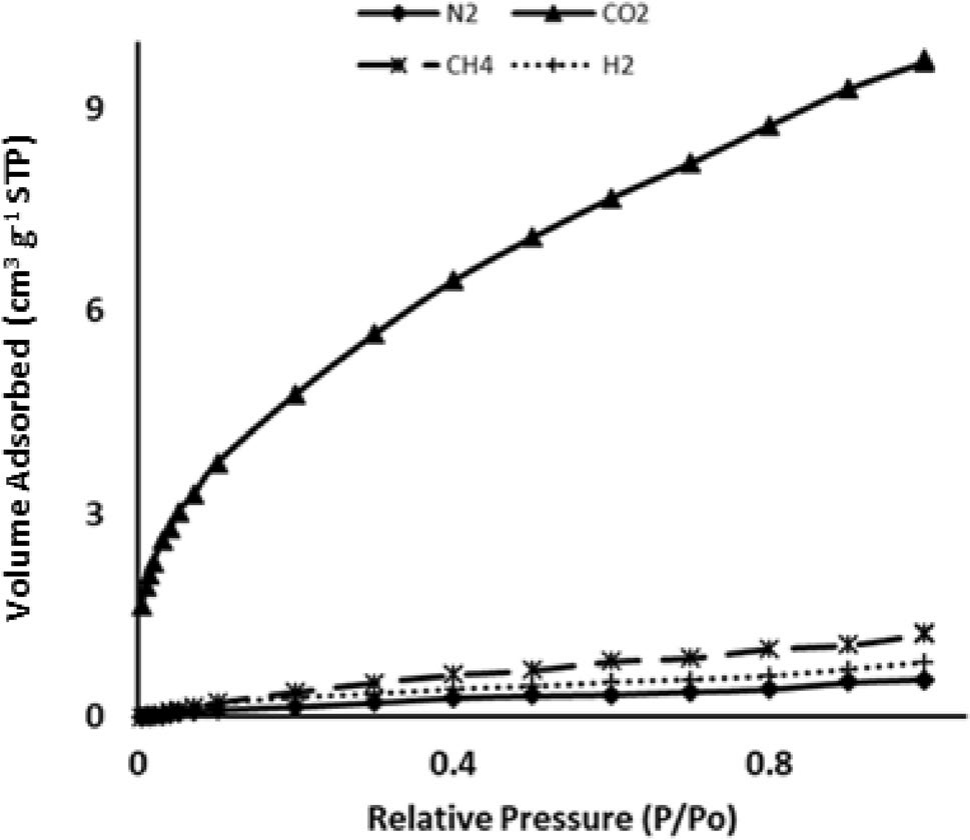
Adsorption isotherm of ASiO_2_ of different gases at 298 K.

**Figure 8:**
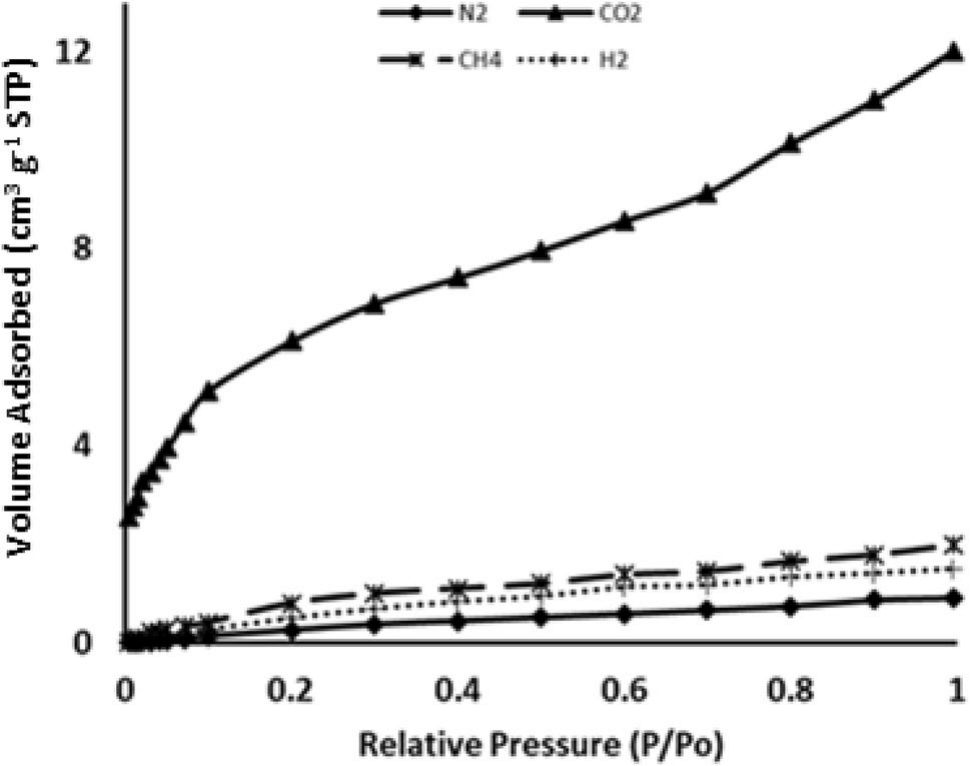
Adsorption isotherm of ASiO_2_ of different gases at 273 K.

**Figure 9:**
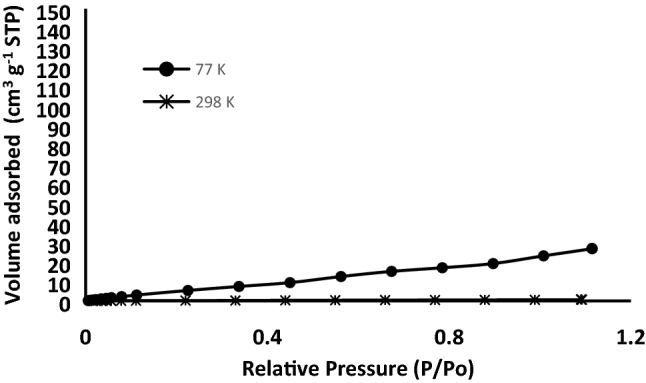
Adsorption isotherm of hydrogen on ASiO_2_ at 298 K and 77 K.

The adsorption isotherms of CO_2_, CH_4_, H_2_, and N_2_ on ASiO_2_ at different temperatures are shown in Figs. 7, 8 and 9. As expected, the adsorption capacity increases with a decrease in temperature. Consistent results were obtained from DFT calculations, which shows an increase in the binding energy as the temperature decreases (Table 2). The results also revealed that ASiO_2_ has a low adsorption capacity of CH_4_, H_2_, and N_2_ while it has an excellent affinity to adsorb CO_2_. Based on the binding energy and bond distance between the adsorbent and adsorbate, the same behaviour was observed in the DFT calculations (Table 2) which revealed a higher affinity of ASiO_2_ to adsorb CO_2_ than CH_4_, H_2_ or N_2_.

**Table 2:** Adsorption energies and binding distances of CO_2_, CH_4_, H_2_ and N_2_ on silica and silica-APTES.

Temperature	Silica			Silica-APTES		
	Gases	ΔE_ads_ (kcal/mol)	Binding distance (Å)	Gases	ΔE_ads_ (kcal/mol)	Binding distance (Å)
298 K	CO_2_	− 8.65	2.891	CO_2_	− 17.6	1.609
	CH_4_	− 7.00	3.233	CH_4_	− 14.5	2.365
	H_2_	− 3.80	4.115	H_2_	− 12.0	2.411
	N_2_	− 6.34	3.746	N_2_	− 9.2	3.176
273 K	CO_2_	− 11.7	2.891	CO_2_	− 29.5	1.505
	CH_4_	− 10.2	2.233	CH_4_	− 20.0	2.032
	H_2_	− 5.04	4.092	H_2_	− 15.6	2.241
	N_2_	− 9.47	3.705	N_2_	− 14.0	2..982
77 K	H_2_	− 25.9	3.562	H_2_	− 63.2	2.056

The adsorption mechanism of CO_2_ by ASiO_2_ can be explained through the formation of ammonium carbonate according to the following equations^21^:

$$ CO_{2} + 2RNH_{2} \to RNHCOO^{ - } + RNH_{3}^{ + } $$

$$ CO_{2} + 2R_{2} NH \to R_{2} NCOO^{ - } + R_{2} NH_{2}^{ + } $$

$$ CO_{2} + R_{2} NH + R^{{\prime }} NH_{2} \to R_{2} NCOO^{ - } + R^{{\prime }} NH_{3}^{ + } $$

The adsorption capacity of RHA for CO_2_ was 0.33 mmol/g while that of ASiO_2_ was 0.43 mmol/g. This improvement in the adsorption capacity due to the amine functional group attached to the silica, as shown in the previous equation. These results are in good agreement with the DFT result that shows a decrease in the energy bandgap between HOMO/LUMO after the functionalization of RHA with the amine group (Fig. 11).

Figure 7 exhibits an excellent gas separation behaviour of ASiO_2_ in which a CO_2_/CH_4_ separation factor (amount adsorbed of CO_2_/amount adsorbed of CH_4_) of 8.6 was achieved, while the separation factors of CO_2_/N_2_ and CO_2_/H_2_ gases were 21.6 and 12.3; respectively^38^.

At 273 K, the adsorption isotherms (Fig. 8) showed the same general behaviour of adsorption affinity with a slight decrease in the separation factors. The separation factors of CO_2_/CH_4_, CO_2_/N_2_, and CO_2_/H_2_ were 6, 13.5, and 7.7, respectively.

Figure 9 showed a good affinity of ASiO_2_ to adsorb H2 at 77 K with an adsorption capacity of 1.2 mmol/g, whereas at 298 K, the adsorption capacity was 0.04 mmol/g. The high binding energy of -63.2 kcal/mol (Table 2) suggests that this behaviour can be related to the formation of hydrogen bonds between hydrogen and silicon at 77 K.

**Figure 10:**
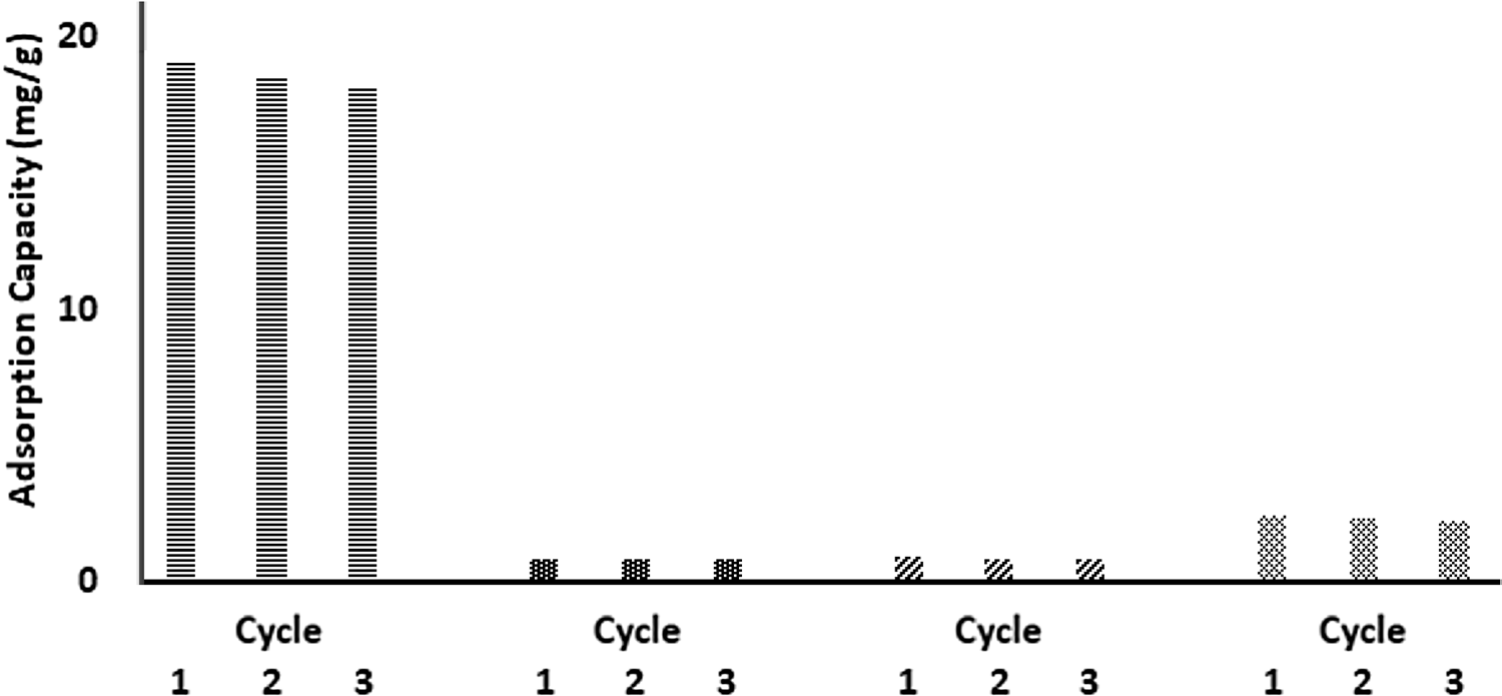
Adsorption recycle of CO_2_, N_2_, CH_4_ at 298 K, and H_2_ at 77 K on ASiO_2_.

The reversibility of gas adsorption was investigated by back-to-back adsorption/desorption cycles, as shown in Fig. 10. The results showed around 5% reduction in the adsorption capacity of CO_2_ between the 1st and 3rd cycles. Besides, the decrease in CH_4_, N_2_, and H_2_ adsorption capacities after the 3rd cycle was about 10%, 6%, and 8%, respectively. This indicates that a simple reduction of the pressure cannot recover a small fraction of the adsorbed molecules; it might also need an elevated temperature to desorb. We believe that if the adsorption/desorption cycles were repeated many more times, the rate of decrease in adsorption capacity would diminish, and the amount adsorbed will be desorbed entirely.

Table 3 shows a collection of data reported on gas adsorption on silica-based materials. Comparable results were noticed for the adsorption capacity of CO_2_ and H_2_. However, higher values were also noticed due to differences in operating temperature or the specific surface area.

**Table 3:** Gas adsorption on different silica-based adsorbents.

Sample	CO_2_ (mmol/g)	CH_4_ (mmol/g)	H_2_ (mmol/g)	N_2_ (mmol/g)	Temp. (K)	Pressure (atm.)	S_BET_ (m^2^/g)	References
*ASiO_2_	0.43	0.05	0.035	0.03	298	1	223	Present
*ASiO_2_	0.54	0.09	0.07	0.04	273	1	223	Present
*ASiO_2_			1.2		77	1	223	Present
APTS-MCM-41	0.54				303	1	198	^15^
SG-APTS	0.9				275	10	270	^19^
MCM-48/TREN	1.5				–	–	263	^20^
MS-400 (25)	3.9				348	1	33	^22^
SBA-PEI	0.8				298	1	5	^21^
SBA-AP	0.85				293	1	562	^18^
APTES-SiO_2_	2.3				273	1	654	^2^
MCM-41			1.2–2.4		77	1	916–1060	^24^
MCF			1.1		77	1	600	^25^
Pt-MCF			1.5–2.5				570–588	
Al-MCF			0.5–1.5				322–498	
SiO_2_			3		77	1	4810	^26^
SiO_2_-OTiCl_3_			1–2				3350–4790	
SBA-15			6–30		77	1	702–3274	^23^
RHA			0.15		77	1	14.05	^43^

### Computational results of gas adsorption

**Figure 11:**
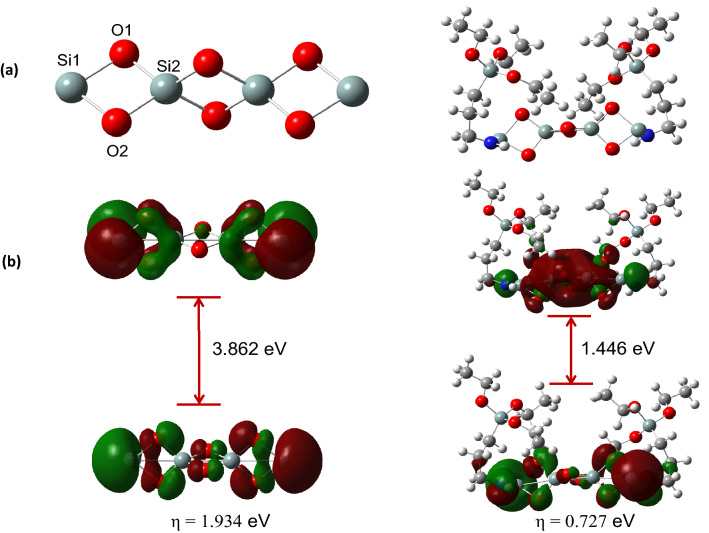
Optimized molecular structures (**a**) and frontier orbital distributions (**b**) of silica cluster, Si_4_O_6_ (left) and silica-APTES (right).

Figure 11 shows the optimized molecular structures of silica, Si_4_O_6_, APTES-functionalized silica, and their frontier orbital distribution. As predicted from the molecular orbital theory, the effectiveness of interaction between two reacting molecules is dependent on their frontier orbitals (HOMO/LUMO) distribution, and the energy gap maintained within^39^. Molecules having lower energy gaps are predicted to exhibit high charge transfer characteristics and are therefore more reactive. Frontier orbital distribution analysis (Fig. 11b) showed that the HOMO–LUMO orbitals were fairly distributed across the silica fragment in both adsorbents. Furthermore, the functionalization of silica with APTES led to a significant decrease in the energy gap from 3.862 eV to 1.446 eV, which consequently implies a substantial increase in reactivity towards the adsorbed gases. Besides, global hardness (η) of the adsorbents, which expresses their tendency to donate their non-bonding electrons during interactions, revealed that the functionalization of silica led to a significant decrease in hardness and a consequent increase in charge transferability.

**Figure 12:**
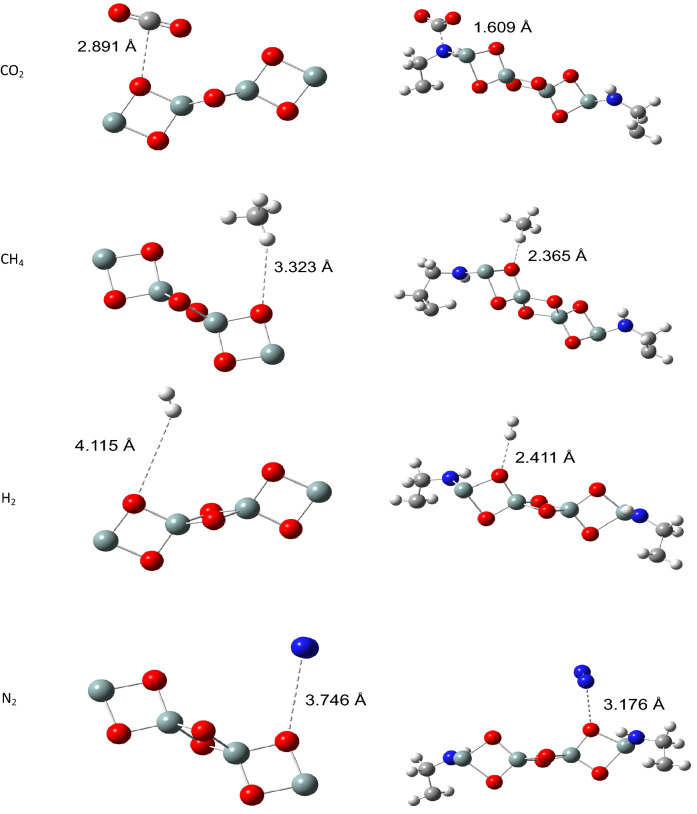
Optimized adsorption of CO_2_, CH_4_, H_2_ and N_2_ on silica, Si_4_O_6_ (left) and silica-APTES (right) at 298 K.

Moreover, interactions of the adsorbents with CO_2_, CH_4_, H_2_, and N_2_ gases were further simulated at 298 K and 273 K, while interactions at 77 K were simulated for H_2_ gas alone. The optimized structures representing the lowest energy conformers of the adsorbed gases onto the adsorbents at 298 K are presented in Fig. 12. Besides, the binding distances between pristine silica and the gases at 298 K; CO_2_ (2.891 Å), CH_4_ (3.233 Å), H_2_ (4.115 Å) and N_2_ (3.746 Å) as opposed to 1.609 Å, 2.365 Å, 2.411 Å, and 3.176 Å in APTES-functionalized silica (supplementary Tables 1-3S) further revealed that the gases only undergo weak physical adsorptions onto pristine silica due to weak van der Waals interactions between the silica surface and the gas molecules which resulted in low adsorption energies. Adsorptions of the gases on APTES-functionalized silica were more favorable with more negative adsorption energies and shorter binding distances due to the increase in surface interactions. Furthermore, functionalized silica showed more affinity to CO_2_ with adsorption energy, Δ*E*_ads_ of − 17.6 kcal/mol (at 298 K), relative to CH_4_ (− 14.5 kcal/mol), H_2_ (− 12.0 kcal/mol) and N_2_ (− 9.2 kcal/mol) gases, and − 29.5 kcal/mol (at 273 K), relative to CH_4_ (− 20.0 kcal/mol), H_2_ (− 15.6 kcal/mol) and N_2_ (− 14.0 kcal/mol) as found from experimental studies. The relatively stronger adsorption of CO_2_ onto APTES-functionalized silica resulting in binding distances 1.609 Å (298 K) and 1.505 Å (273 K) could be due to the enhancement in basicity character of the adsorbent upon functionalization which led to chemical interaction (chemisorption) with CO_2_ as implied by the binding distances^40,41^, and in accordance with the hard-soft acid-base (HSAB) principle^42^. Overall, DFT results are in good correlation with experimental data and showed that the functionalization of silica with APTES led to an increase in adsorption efficiency, and adsorption efficiency increases with a decrease in temperature.

## Conclusions

The utilization of agricultural waste material (Rice Husk) was successfully achieved to prepare silica (RHA) which was functionalized with APTES to get aminated silica. Quantum chemical DFT calculations revealed that the functionalization of RHA to produce (ASiO_2_) resulted in a decrease in the HOMO–LUMO energy gap, a corresponding reduction in hardness, an increase in charge transfer characteristics and consequently higher interactions with the studied gases. The adsorption capacity of the gases on ASiO_2_ increased in the order CO_2_ > CH_4_ > H_2_ > N_2_, which was consistent with the DFT calculations in terms of adsorption energy and binding distance. Aminated silica showed a good separation factor of CO_2_ from the other studied gases at 298 K. The separation factor of CO_2_/N_2_ and CO_2_/CH_4_ at 298 K was 21.5 and 8.6, respectively. The separation factor of ASiO_2_ towards binary mixture of the gases makes it a suitable candidate for applications in natural gas separation as well as in environmental applications.

## Supplementary information

Supplementary Information.
