# Supplementary material for: Rice husk derived Aminated Silica for the efficient adsorption of different gases
Source: Sci Rep. 2020 Nov 11;10:19526. doi: 10.1038/s41598-020-76460-0 (PMC7658350; doi:10.1038/s41598-020-76460-0)
Supplement: Supplementary file 1 — Supplementary Information. [file 41598_2020_76460_MOESM1_ESM.docx]

**Supplementary Material:**

**Rice husk derived Aminated Silica for the efficient adsorption of different gases**

Rashed S. Bakdash^1^, Isam. H. Aljundi^2*^, Chanbasha Basheer^1^*, Ismail Abdulazeez^1^

^1^Department of Chemistry, King Fahd University of Petroleum and Minerals, Dhahran 31261, Saudi Arabia

^2^Department of Chemical Engineering, King Fahd University of Petroleum and Minerals, Dhahran 31261, Saudi Arabia


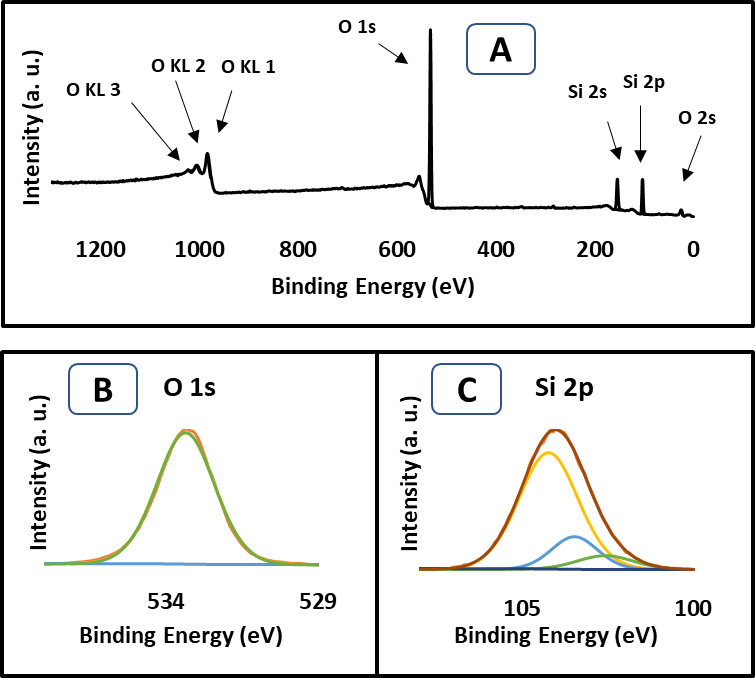


Fig. S1 XPS analysis of RHA, A: wide scan spectrum, high resolution spectrum of B: O 1s and C: Si 2p

X-ray photoelectron spectroscopy (XPS), comprehensive scan (Fig. S1-A) of RHA, shows the presence of oxygen and silicon on the surface, which confirms the successful preparation of SiO_2_ from rice husk. The high-resolution scans reveal the chemical state and types of bonding present in the elements. In Fig S1-B, a band was noticed in the high-resolution scan of (O 1s) at ∼533.3 eV that can be accredited to the Si-O bond of silica [1]. The data in (Fig. S1-C) demonstrates the high-resolution XPS band of Si (2p). The deconvoluted binding energy peaks at ∼103 and 104 eV can be ascribed to the Si-O bonds with different oxidation state (Si^4+^) and (Si^3+^) while the peak at ∼102 can be attributed to Si-Si bond with oxidation stat of (Si^2+^) [1,2], [3].

Table S1: Bond properties of selected atoms upon adsorption of CO_2_, CH_4_, H_2_ and N_2_ on silica and silica-APTES at 298 K

| **Adsorbent/gas** | **Bond** | **Bond distance (Å)^a^** | **Angle** | **Bond angle (^o^)^b^** |
| --- | --- | --- | --- | --- |
| Silica/CO_2_  Silica/CH_4_  Silica/H_2_  Silica/N_2_ | Si1-O1  Si1-O2  Si2-O1  Si2-O2  Si1-O1  Si1-O2  Si2-O1  Si2-O2  Si1-O1  Si1-O2  Si2-O1  Si2-O2  Si1-O1  Si1-O2  Si2-O1  Si2-O2 | 1.837 (1.835)  1.828 (1.835)  1.770 (1.769)  1.771 (1.769)  1.835 (1.835)  1.835 (1.835)  1.769 (1.769)  1.769 (1.769)  1.835 (1.835)  1.835 (1.835)  1.769 (1.769)  1.769 (1.769)  1.835 (1.835)  1.835 (1.835)  1.769 (1.769)  1.769 (1.769) | Si1-O1-Si2  Si1-O2-Si2  O1-Si1-O2  O1-Si2-O2  Si1-O1-Si2  Si1-O2-Si2  O1-Si1-O2  O1-Si2-O2  Si1-O1-Si2  Si1-O2-Si2  O1-Si1-O2  O1-Si2-O2  Si1-O1-Si2  Si1-O2-Si2  O1-Si1-O2  O1-Si2-O2 | 94.0 (93.8)  94.3 (93.8)  84.0 (93.8)  87.7 (93.8)  93.8 (93.8)  93.8 (93.8)  84.2 (93.8)  88.2 (93.8)  93.8 (93.8)  93.8 (93.8)  84.3 (93.8)  88.2 (93.8)  93.8 (93.8)  93.8 (93.8)  84.3 (93.8)  88.2 (93.8) |
| Silica-APTES/CO_2_  Silica-APTES/CH_4_  Silica-APTES/H_2_  Silica-APTES/N_2_ | Si1-O1  Si1-O2  Si2-O1  Si2-O2  Si1-O1  Si1-O2  Si2-O1  Si2-O2  Si1-O1  Si1-O2  Si2-O1  Si2-O2  Si1-O1  Si1-O2  Si2-O1  Si2-O2 | 1.885 (1.905)  2.057 (2.042)  1.743 (1.736)  1.723 (1.726)  1.914 (1.905)  2.031 (2.042)  1.738 (1.736)  1.726 (1.726)  1.915 (1.905)  2.030 (2.042)  1.738 (1.736)  1.726 (1.726)  1.909 (1.905)  2.038 (2.042)  1.738 (1.736)  1.726 (1.726) | Si1-O1-Si2  Si1-O2-Si2  O1-Si1-O2  O1-Si2-O2  Si1-O1-Si2  Si1-O2-Si2  O1-Si1-O2  O1-Si2-O2  Si1-O1-Si2  Si1-O2-Si2  O1-Si1-O2  O1-Si2-O2  Si1-O1-Si2  Si1-O2-Si2  O1-Si1-O2  O1-Si2-O2 | 97.2 (95.9)  91.8 (91.4)  78.5 (79.2)  92.3 (93.4)  95.7 (95.9)  92.0 (91.4)  79.1 (79.2)  93.1 (93.4)  95.7 (95.9)  92.0 (91.4)  79.1 (79.2)  93.0 (93.4)  95.8 (95.9)  91.2 (91.4)  79.2 (79.2)  93.2 (93.4) |

^a^ Values in parenthesis represents bond distances in isolated silica and silica-APTES

^b^ Values in parenthesis represents bond angles in isolated silica and silica-APTES

Table S2: Bond properties of selected atoms upon adsorption of CO_2_, CH_4_, H_2_ and N_2_ on silica and silica-APTES at 273 K

| **Adsorbent/gas** | **Bond** | **Bond distance (Å)^a^** | **Angle** | **Bond angle (^o^)^b^** |
| --- | --- | --- | --- | --- |
| Silica/CO_2_  Silica/CH_4_  Silica/H_2_  Silica/N_2_ | Si1-O1  Si1-O2  Si2-O1  Si2-O2  Si1-O1  Si1-O2  Si2-O1  Si2-O2  Si1-O1  Si1-O2  Si2-O1  Si2-O2  Si1-O1  Si1-O2  Si2-O1  Si2-O2 | 1.837 (1.835)  1.828 (1.835)  1.771 (1.769)  1.771 (1.769)  1.836 (1.835)  1.835 (1.835)  1.769 (1.769)  1.769 (1.769)  1.835 (1.835)  1.835 (1.835)  1.769 (1.769)  1.769 (1.769)  1.835 (1.835)  1.835 (1.835)  1.769 (1.769)  1.769 (1.769) | Si1-O1-Si2  Si1-O2-Si2  O1-Si1-O2  O1-Si2-O2  Si1-O1-Si2  Si1-O2-Si2  O1-Si1-O2  O1-Si2-O2  Si1-O1-Si2  Si1-O2-Si2  O1-Si1-O2  O1-Si2-O2  Si1-O1-Si2  Si1-O2-Si2  O1-Si1-O2  O1-Si2-O2 | 94.0 (93.8)  94.3 (93.8)  84.0 (93.8)  87.7 (93.8)  93.8 (93.8)  93.8 (93.8)  84.2 (93.8)  88.2 (93.8)  93.8 (93.8)  93.8 (93.8)  84.3 (93.8)  88.2 (93.8)  93.8 (93.8)  93.8 (93.8)  84.3 (93.8)  88.2 (93.8) |
| Silica-APTES/CO_2_  Silica-APTES/CH_4_  Silica-APTES/H_2_  Silica-APTES/N_2_ | Si1-O1  Si1-O2  Si2-O1  Si2-O2  Si1-O1  Si1-O2  Si2-O1  Si2-O2  Si1-O1  Si1-O2  Si2-O1  Si2-O2  Si1-O1  Si1-O2  Si2-O1  Si2-O2 | 1.924 (1.905)  2.021 (2.042)  1.743 (1.736)  1.724 (1.726)  1.907 (1.905)  2.046 (2.042)  1.738 (1.736)  1.725 (1.726)  1.914 (1.905)  2.030 (2.042)  1.739 (1.736)  1.726 (1.726)  1.909 (1.905)  2.038 (2.042)  1.738 (1.736)  1.726 (1.726) | Si1-O1-Si2  Si1-O2-Si2  O1-Si1-O2  O1-Si2-O2  Si1-O1-Si2  Si1-O2-Si2  O1-Si1-O2  O1-Si2-O2  Si1-O1-Si2  Si1-O2-Si2  O1-Si1-O2  O1-Si2-O2  Si1-O1-Si2  Si1-O2-Si2  O1-Si1-O2  O1-Si2-O2 | 95.7 (95.9)  92.9 (91.4)  78.7 (79.2)  92.4 (93.4)  95.9 (95.9)  91.4 (91.4)  79.1 (79.2)  93.4 (93.4)  95.7 (95.9)  92.0 (91.4)  79.1 (79.2)  93.0 (93.4)  95.8 (95.9)  91.6 (91.4)  79.2 (79.2)  93.2 (93.4) |

^a^ Values in parenthesis represents bond distances in isolated silica and silica-APTES

^b^ Values in parenthesis represents bond angles in isolated silica and silica-APTES

Table S3: Bond properties of selected atoms upon adsorption of H_2_ on silica and silica-APTES at 77K

| **Adsorbent/gas** | **Bond** | **Bond distance (Å)^a^** | **Angle** | **Bond angle (^o^)^b^** |
| --- | --- | --- | --- | --- |
| Silica/H_2_ | Si1-O1  Si1-O2  Si2-O1  Si2-O2 | 1.826 (1.835)  1.835 (1.835)  1.769 (1.769)  1.769 (1.769) | Si1-O1-Si2  Si1-O2-Si2  O1-Si1-O2  O1-Si2-O2 | 92.5 (93.8)  93.8 (93.8)  84.3 (93.8)  88.2 (93.8) |
| Silica-APTES/H_2_ | Si1-O1  Si1-O2  Si2-O1  Si2-O2 | 1.911 (1.905)  2.030 (2.042)  1.739 (1.736)  1.726 (1.726) | Si1-O1-Si2  Si1-O2-Si2  O1-Si1-O2  O1-Si2-O2 | 93.7 (95.9)  92.0 (91.4)  79.1 (79.2)  93.0 (93.4) |

^a^ Values in parenthesis represents bond distances in isolated silica and silica-APTES

^b^ Values in parenthesis represents bond angles in isolated silica and silica-APTES

Bond properties of the isolated adsorbents and the adsorbent-gases complexes at 298 K are presented in Table S1, while those at 273 K and 77 K (for H_2_ alone) are given in Table S2 and Table S3. It can be seen from Table S1 that while no significant changes in the bond properties of pure silica were obtained as a result of adsorption of the gases; slight changes were visible in the APTES-functionalized silica due to enhancement in charge transfer characteristics which resulted in enhanced interactions with the gases. For instance, the Si1-O1 bond was shorter in the CO_2_ adsorbed complex (1.885 Å) compared to the isolated adsorbent (1.905 Å), while the Si1-O2 bond was elongated (from 2.042 Å to 2.057 Å). The bond angles Si1-O1-Si2 also increased from 95.9 deg. in the isolated adsorbent to 97.2 deg. in the CO_2_ adsorbed complex.

**References:**

[1] Li M, Zhuang L, Wang X, Shen H, Zeng L, Chen Y. Realization of Colored Multicrystalline Silicon Solar Cells with SiO 2 /SiN x :H Double Layer Antireflection Coatings . Int J Photoenergy 2013;2013:1–8. https://doi.org/10.1155/2013/352473.

[2] Ghita R, Logofatu C, Negrila C-C, Ungureanu F, Cotirlan C, Manea A-S, et al. Study of SiO2/Si Interface by Surface Techniques, Crystalline Silicon - Properties and Uses, InTech; 2011, p. 23–42. https://doi.org/10.5772/23174.

[3] Choi M, Kim J-C, Kim D-W. Waste Windshield-Derived Silicon/Carbon Nanocomposites as High-Performance Lithium-Ion Battery Anodes. Sci Rep 2018;8:960. https://doi.org/10.1038/s41598-018-19529-1.
